# Effect of Self-Care Education by Face-to-Face Method on the Quality of Life in Hemodialysis Patients *(Relying on Ferrans and Powers Questionnaire)*

**DOI:** 10.5539/gjhs.v8n6p121

**Published:** 2015-10-20

**Authors:** Mahsa Sabet Ghadam, Farzad Poorgholami, Zohreh Badiyepeymaie Jahromi, Nehleh Parandavar, Navid Kalani, Elham Rahmanian

**Affiliations:** 1 Department of Obstetrics and Gynecology, Jahrom University of Medical Sciences, Jahrom, Iran; 2 Reaserch center for non-Communicable Diseases, Jahrom University of Medical Sciences, Jahrom, Iran; 3 Department of Nursing and Midwifery, Jahrom University of Medical Sciences, Jahrom, Iran; 4 Department of Student Research Committee, Jahrom University of Medical Sciences, Jahrom, Iran; 5 Cellular and Molecular Gerash research center, Grash School of Medical Science, Shiraz University of Medical Science, Shiraz, Iran

**Keywords:** self-care, face-to-face, quality of life, hemodialysis

## Abstract

**Introdution:**

One of the most common methods to control chronic renal failure, Hemodialysis creates numerous changes in the style and the quality of life in patients. Educating patients is one of effective factors to improve the quality of life. The present study aims to investigate influences of self-care education by face-to-face method on determining quality of life in hemodialysis patients in Jahrom, Iran, during 2014-2015.

**Methods:**

This is a quasi-experimental, single-blind study in which 50 patients undergoing hemodialysis at Shahaid Mottahari Hospital, Jahrom. The patients were placed in two groups of 25 individuals: the face to face educational group and the control group. The control group received only routine care in hemodialysis unit. The face to face educational group received 8 instruction sessions of 60 minutes before starting dialysis and received an instruction booklet. Data collection tools were a questionnaire consisting of demographic characteristics, a checklist of needs assessment for hemodialysis patients and a quality of life questionnaire, whose reliability and validity were previously approved. The questionnaires were completed face to face, before and after the intervention.

**Results:**

The results show that the research units did not have any significant difference in terms of demographic variables. Also increase in various aspects of the quality of life compared with the control group is observed after the intervention in the face to face educational group (p<0.001).

**Discussion and Conclusion:**

Given the results, representation of adequate training in hemodialysis ward can cause improve in physical function, mental health and thus increase the quality of life in hemodialysis patients, through raising the awareness level.

## 1. Introduction

Increasing prevalence of chronic disease is the most prominent event in this century that communities and health staff have faced with it and habits and health behaviors in individuals dramatically impact on its prevalence (Smeltzer. et al, 2008). End stage renal failure is a chronic condition (Bard, 2010) and occurs when 95 percent of kidneys’ function is lost. Treatment methods of the disease are including long-term dialysis or kidney transplant (Tsay. et al, 2002). Nowadays, a large number of people in Iran are suffering from kidney diseases and they have multiple problems in terms of medical and treatment cost in health organizations. Now, more than 17,000 patients with kidney failure live in Iran that most of them are regularly controlled with dialysis and this number has increased significantly compared to the previous years (Poorgholami et al, 2016).

Hemodialysis patients experience many problems including sleep disorders, peripheral neuropathy, infection, psychological stress, anxiety and depression, cognitive changes, wanness, reduction of viscosity and so on (Lee, 1997; Domrongkitchaiporn et al., 2005; Akyuz et al., 2009; Kargar Jahromi et al., 2016; Brenner et al., 2008; Urden et al., 2006). Various problems affect on different aspects of life in patients undergoing hemodialysis. Also several studies have shown that patients undergoing hemodialysis have lower quality of life in compared to other normal individuals in society (Finkelstein et al., 2009; Kimmel et al., 2006). Hemodialysis can seriously affect people’s quality of life and it can disrupt the amount of physical and social activity and life satisfaction in patients (Coelho et al., 2003).

Due to the long-term process of hemodialysis treatment, these patients necessarily need to change their lifestyle in order to better cope and to manage their disease (Cowen, 2006). In addition to adaptation to renal disease, these patients should also compromise with multiple stresses from their spouse, family, job and society (Kimmel. et al., 2006). Each dialysis time was performed an average four hours and three times in a week and most of patients were suffered from fatigue, muscle cramps and headache after dialysis. These patients were forced to adapt to all of these conditions (Pakpour, 2011). Quality of life is a multidimensional and comparative concept that is affected by time, location, and social and personal values (Ioannidis, 2001). Quality of life is something more than health condition because health is just one dimension of it and the other factors affecting the quality of life are including one’s own satisfaction, family, social and economical statuses and most importantly mental and emotional statuses (Ferrans, 1992).

A complex care is needed for chronic kidney diseases (Neyhart et al., 2010). Aim of care is idealizing quality of life in patients and application of nursing theories and models is a way for modification of it (Rahimi et al., 2006). Having a model of nursing care in hemodialysis ward is completely essential to support patients’ individual needs, to ensurestandard nursing care and to maintain quality of care (Dobson et al., 2008).

Treatment of hemodialysis patients cannot be effective enough and desired treatment results cannot be achieved without participation of the patients themselves and implementation of self-care activities (Cowen, 2006). Appropriate training programs on disease, treatment method and cases that should be obeyed by patient (self-care) improve physical function and general health as well as emotional, psychological and social statuses of patients (Rafie Fard et al., 2008). Self-care as a regulatory function is different from other regulatory functions of human such as neuroendocrine, since self-care must be learned, and it must performed as a conscious and continuous activity in accordance with regulatory needs of individuals (Meleis, 2005). The purpose of self-care measures is establishment and/or continuation of necessary materials for growth and health promotion. These measures include prevention, relieve of pain, cure or control of disease and health life-threatening condition (Meleis, 2005). In general, self-care measures improve quality of life in patients (Oshvandi, 1995).

Quality of life improvement is generally considered as one of the main objectives in treatment of chronic patients (Kasper, 2005) and due to increasing rate of chronic kidney disease as well as high cost of treatment and quality of life losses in hemodialysis patients, using low-cost and appropriate treatment appears to be necessary to improve quality of life in these patients. So according to above, the purpose of the research is to evaluate the effect of self-care education by face-to-face method on quality of life level in hemodialysis patients and perhaps in this way can help to improve quality of life in these patients that constitute a large segment of populations.

## 2. Methods

This quasi-experimental, pretest-posttest interventional study was conducted in the dialysis units in Mottahari Hospital affiliated with the Jahrom University of Medical Sciences, Jahrom, Iran during 2014-2015. These hospitals were governmental referral centers. After obtaining necessary permissions and coordinating with authorities of hemodilysis ward, 50 hemodilysis patients who were eligible for inclusion to the research were selected as samples through a purposive sampling and then were randomly divided into a control group and face to face educational group. The patients were placed in two groups of 25 individuals. Inclusion criteria for this study included age of 18-65 years, having at least the ability to read and write, not having hearing and vision impairments, being at least 6 months on hemodialysis, and not participating in any educational class out of the routine ones for at least during the last year. Exclusion criterion was not undergoing kidney transplant surgery during the study.

Data collection tools were included demographic questionnaire, hemodialysis patients’ checklist of requirements, and quality of life questionnaire. An assessing checklist for requirements of hemodialysis patients was used to study problems and requirements of patients (Moattari, 2012). This tool is designed to help nurses in finding of training requirements and is completed by researcher in an interview. Patient problems according to the checklist were investigated in 7 general areas include: medical and nutritional cases relationship with family and friends, responsibility against disease, patients’ future, life style and daily activities, and communications with hospital staffs (Moattari, 2012).

Ferrans and Powers quality of life questionnaire (dialysis version): this questionnaire was made by Carol Estwing Ferrans and Marjorie Powers, Illinois Chicago Faculty of Nursing professors, (1986) to measure quality of life in terms of life satisfaction. Quality of life is measured in two parts, satisfaction and importance.

The questionnaire was consisted of two parts. The first part measured satisfaction with various aspects of life and the second measured the importance of the same aspects importance ratings were used to weigh the satisfaction responses. Therefore, the scores reflected the respondents´satisfaction with the aspects of life as they value. Items rated as more important have a greater impact on scores than those of lesser importance. The scores were calculated both for overall quality of life and four subscales of health and functioning; psychological/spiritual; social and economic; and family. The quality of life can be used either as a self-administered questionnaire or through an interview. If self-administered, the quality of life took approximately ten minutes to complete. No special training was required. The validity and reliability of this questionnaire was reported by Ferrans and Powers in 1985 and 1992 (Moattari, 2012). This questionnaire was translated into Persian by Rambod and its validity and reliability have been confirmed (Rambod et al., 2009). The possible range for the final scores is the same for all four subscales and for the overall (total) score Total scores may be classified in three levels: desirable (score: 10 - 19) and unfavorable (score: 0 - 9). Data collection process was performed as follows; after the entrance of qualified patients according to the criteria of entrance to the study, demographic questionnaire and the assessment checklist of hemodialysis patients were completed by the researcher and the co-researcher in separate interviews. Then a quality of life questionnaire was given to each patient to fill it out before the intervention. The intervention method was so that there was no intervention for the control group and patients received only routine hospital care. Hemodialysis patients in the face to face education group were trained by the researcher in approximately one hour face-to-face training for eight sessions before starting dialysis. Conversation topics in face-to-face training included, understanding the disease process, hemodialysis importance, diet, limitation for food intake, body weight control, physical activity, control of vital signs, familiarity with the symptoms of underlying the disease, importance of stopping smoking, stress management and muscular relaxation. Patients also received a researcher-made booklet. The results were analyzed by SPSS version 20. The data were examined using Chi-square, independent t-test and Paired t-test.

## 3. Results

Chi-square test showed that both groups are similar in socio demographic characteristics. Table 1 shows some socio-demographic characteristics of the patients.

**Table 1:** Frequency distribution of the study units based on demographic variables in two groups

Group Features		Face to face education	Control	P value

		Relative frequency	Relative frequency	
Sex	Female	56%	40%	0.42
	Male	44%	60%	
Marital status	Single	28%	16%	0.27
	Married	72%	84%	
Employment	Yes	40%	56%	0.42
	No	60%	44%	
Education level	Primary	4%	4%	0.63
	Junior high school	36%	36%	
	High school	56%	48%	
	Academic	4%	12%	
Hemodialysis frequency in a week	Twice	44%	28%	0.17
	Three times	56%	72%	
	Poor	36%	20%	0.32
Income level	Average	44%	72%
	Good	20%	8%

The mean of quality of life scores in hemodialysis patients before intervention in each of the two groups are shown in Table 2. Using independent sample t-test, no significant difference was observed between the two groups in all dimensions of quality of life questionnaire before intervention.

**Table 2:** Mean of quality of life scores before the intervention in the two groups

Groups	Face to face Education	Control	P- value
	
Quality of Life Aspects	Standard Deviation and Mean	Standard Deviation and Mean	
Health of Functionality	12.074	11.981	P=0.4
	0.398	0.426	
Social-Economical	12.175	12.187	P=0.5
	0.415	0.479	
Mental – Spiritual	11.800	15.297	P=0.4
	0.635	0.770	
Family	13.232	13.084	P=0.4
	0.843	0.847	
Total Score	12.196	12.141	P=0.4
	0.316	0.294	

*Note.* significance level considered by P<05.

Significant differences were observed between the two groups in the posttest regarding the dimensions scores of quality of life questionnaire. The mean scores of the intervention group were higher than those of the controls (Table 3)

**Table 3:** Mean of quality of life scores after the intervention in the two groups

Groups	Face to face Education	Control	P- value
	
Groups Quality of Life Aspects	Mean and Standard Deviation	Mean and Standard Deviation	
Health of Functionality	15.062	12.090	P=0.001
	0.542	0.444	
Social-Economical	14.707	12.160	P=0.001
	0.882	0.434	
Mental – Spiritual	11.737	11.725	P=0.001
	1.121	0.703	
Family	14.256	13.256	P=0.001
	0.591	0.841	
Total Score	14.256	12.218	P=0.001
	0.290	0.289	

*Note.* significance level considered by P<05.

## 4. Discussion

The results show that characteristics or properties of the subjects such as age, gender, education level, marital status, employment status, level of income, number of dialysis per week, and duration of dialysis by hemodialysis did not have a significant difference at the level of 5% in both groups, in other words the research unites were equally distributed in the studied groups.

In studies that till now performed in the field of renal progressive failure and hemodialysis in Iran, assessment of quality of life in hemodialysis patients, stress and stress adjustment mechanisms with quantitative or statistical methods have been further investigated. The results showed that both groups before intervention were the same in terms of background variables. Dimensions of quality of life were measured at the beginning of the study in both groups (control and educational). The results showed that a significant percentage of hemodialysis patients in both control and educational groups had low level in quality of life.

Orlandi FS in Sao Paulo, Brazil (2012) performed a research entitled evaluation of hope in elderly patients undergoing hemodialysis and stated that men constituted 60 percent of participants in his study (Fatehi et al., 2013). Also in the current study percentage of men was statistically more than women (in total of both groups (control and educational)). Orlandi FS stated that men had greater chance for getting chronic renal failure (Fatehi et al., 2013).

The results of the research corresponded to research of Narimani et al., in 2008 that reported quality of life increased (including psychological aspect (p=0.002)) in hemodialysis patients following a self-care education program (Fatehi et al., 2013). Pandora Self-Efficacy Theory is based on an assumption that judgment of person about self abilities can cause application of self-care behaviors in order to achieve the desired results. The judgment or self-efficacy is a bridge between knowledge and real self-care behaviors. Increasing of self-care knowledge with increasing acceptance of treatment has caused to perform self-care behaviors (Fatehi et al., 2013). Tesai quoted from Lavon that there is a positive relation between responsibility in self-care and quality of life (Fatehi et al., 2013).

Yen et al. stated that education increased physical, mental and social aspects of their patients (Yen et al., 2008). Also a research by Baraz et al. confirmed this result (Baraz et al., 2005). Mental aspects of quality of life before and after intervention in the experimental group in the current study were 11.725 and 15.297 respectively, which represented an increase in mental aspect after performance of the self-care educations in dialysis patients. The obtained results were consistent with previous researches that represented positive effects of self-care education in mental and social aspects of quality of life in the current research.

It was shown in the past that self-care training was effective for one year on quality of life in patients undergoing hemodialysis and this type of training increased different aspects of quality of life in patients undergoing hemodialysis (Loos-Ayav et al, 2008). Also in the current study, increase of quality of life in the educational group was observed after performance of the training. This was consistent with previous research.

Studies suggested that people, who believed in their performance and health of functionality, were doing additional efforts to overcome problems and obstacles. Lee et al. found that self-efficacy and health of functionality score in the intervention group increased than the control group (Lee et al., 2006). A research by Zaman Zadeh et al. showed that education increased patients’ effort to obtain social supports, also increased motivation and decision making power in addition to improve self-efficacy and health of functionality (Zamanzdeh et al., 2008).

Paying attention to control threatening situations and to increase self-efficacy and health of functionality is very important for treatment of undergoing hemodialysis patients who live in special circumstances (Soultani Nejad, 2014). Santos in his study in Brazil found that quality of life in hemodialysis patients has gone higher by using of training methods of effective confronting (Santos, 2010). Past studies that performed on hemodialysis patients, in Bushehr (2013-2014), showed that presentation of adequate training in hemodialysis ward by increasing awareness level in patients created public health, physical functioning and mental health improvement, and created general perception of health and thus enhance quality of life in hemodialysis patients. The mentioned results were in consistent with the obtained results from the current research, as comparison of means after self-care education in experimental groups showed an increase in different dimensions of quality of life in hemodialysis patients compared to before self-care education.

## 5. Conclusions

Implementation of the self-care training program according to the findings of this research is effective to increase dimensions of quality of life in patients undergoing hemodialysis. Also self-care training improves functionality of patients. Therefore, to enhance physical and mental health in hemodialysis patients continuous training in the field of self-care seems to be effective.
